# Burnout Syndrome Among Dental Students in Clinical Training: A Multicenter Cross-Sectional Study in Ecuador

**DOI:** 10.3390/ijerph22091393

**Published:** 2025-09-06

**Authors:** Luis Chauca-Bajaña, Andrea Ordoñez Balladares, Ivonne Alison Carrión Bustamante, Andrea Carolina Sánchez Salcedo, Juan Suárez-Palacios, Xavier Andrés Villao-León, Francisco Jorge Morán Peña, Rita Carolina Egüés Cevallos, Roberto Tolozano-Benites, Byron Velásquez Ron

**Affiliations:** 1 Dental Sciences, College Dentistry, University of Guayaquil, Guayaquil 090514, Ecuador; luis.chaucab@ug.edu.ec (L.C.-B.); andrea.ordonezb@ug.edu.ec (A.O.B.); 2 Dental Sciences, College Dentistry, Universidade Santiago de Compostela, 15782 Santiago de Compostela, Spain; 3 Dental Sciences, College Dentistry, Bolivarian University of Ecuador, Durán 092406, Ecuador; 4 Esthetic and Operative Dentistry, College Dentistry, University of Guayaquil, Guayaquil 090514, Ecuador; ivonne.carrionb@ug.edu.ec; 5 Oral Rehabilitation, College Dentistry, University of Guayaquil, Guayaquil 090514, Ecuador; andrea.sanchezs@ug.edu.ec (A.C.S.S.); juan.suarezpa@ug.edu.ec (J.S.-P.); 6 Endodontics Oral Research, College of Dentistry, University of Guayaquil, Guayaquil 090514, Ecuador; xavier.villaol@ug.edu.ec; 7 Educational Informatics, College of Dentistry, University of Guayaquil, Guayaquil 090514, Ecuador; jorge.moranp@ug.edu.ec (F.J.M.P.); rita.eguezc@ug.edu.ec (R.C.E.C.); 8 Pedagogical Sciences Education, Universidad Bolivariana del Ecuador, Durán 092406, Ecuador; rtolozano@ube.edu.ec; 9 Dental Prosthesis Department Research, College Dentistry, University of the Americas, UDLA. Av, Colon y 6 de diciembre, Campus Colón, Quito 170102, Ecuador

**Keywords:** burnout syndrome, dental students, mental health

## Abstract

Burnout syndrome, caused by chronic unmanaged stress, is common among health sciences students, and dental students in clinical training are particularly vulnerable due to the intense cognitive, emotional, and practical demands. This multicenter cross-sectional study assessed burnout and related factors in 312 students in their 8th–10th semesters at three Ecuadorian universities using the Maslach Burnout Inventory and a questionnaire on physical and emotional symptoms. High emotional exhaustion affected 79.5% of students, high depersonalization 54.5%, and low personal accomplishment 11.5%, with an overall burnout prevalence of 8.01%. No statistically significant associations were found with university or academic semester, although students studying or working more than 30 h per week showed a non-significant trend toward higher risk (OR = 3.39; *p* = 0.208), and the model’s predictive capacity was low (AUC = 0.645). Frequently reported physical symptoms included lower back pain (41.35%), neck pain (35.9%), and headaches (30.45%). These findings reveal that burnout, often accompanied by physical discomfort, affects a significant number of dental students, highlighting the need for institutional strategies such as psychological support and curricular adjustments to reduce stress and improve overall well-being.

## 1. Introduction

Burnout syndrome has been recognized by the World Health Organization (WHO) as a significant public health concern. It is included in the 11th edition of the International Classification of Diseases (ICD-11) as a condition resulting from chronic workplace stress that has not been successfully managed [1,2]. This syndrome is characterized by physical, emotional, and behavioral symptoms, reflecting an advanced state of exhaustion. Its core components include emotional exhaustion, depersonalization, and reduced personal accomplishment, which can profoundly impact individuals’ well-being and their performance in activities that require sustained commitment [3].

The term burnout was introduced by Herbert Freudenberger in 1974, describing it as a progressive depletion resulting from chronic stress, particularly among individuals engaged in tasks that failed to provide the expected sense of fulfillment [4]. Later, in 1976, Christina Maslach expanded on this concept at the University of California, Berkeley, by developing a three-dimensional model that includes emotional exhaustion, depersonalization, and reduced personal accomplishment [5,6,7]. Based on this theoretical framework, various tools were developed to assess the syndrome, with the Maslach Burnout Inventory (MBI) being the most widely used and internationally validated instrument in both occupational and academic settings [8,9]. Although burnout was initially associated with professionals in the healthcare or education sectors, it is now recognized that it can affect anyone who is continuously exposed to high levels of emotional demand, as is often the case with university students [10,11]. In particular, the field of Dentistry represents a particularly vulnerable environment for the development of this syndrome, due to the high cognitive, emotional, and practical demands it entails [12]. During their clinical training, dental students face multiple challenges: direct responsibility for patient care, pressure to achieve satisfactory clinical outcomes, academic competition, long practical sessions, and exposure to emotionally demanding situations. This combination of factors creates a conducive environment for exhaustion and the onset of burnout symptoms [13]. Previous studies have shown that students in the health sciences, particularly those in Medicine and Dentistry, exhibit high levels of distress, psychological strain, and even psychiatric disorders associated with sustained academic stress [14,15,16]. A study conducted by Sravan et al. (2018) involving 159 dental students, the majority of whom were women, reported a significant prevalence of emotional exhaustion (39.6%), high depersonalization (46.5%), and low levels of personal accomplishment in 78.6% of the participants [17]. These findings reflect a concerning trend that has been supported by multiple studies, which indicate that both individual factors (such as coping strategies and resilience) and contextual factors (such as the institutional environment and faculty support) influence the development of the syndrome [18]. Moreover, burnout has been observed to coexist with disorders such as anxiety and depression, affecting between 10% and 43.8% of the student population, with particularly high prevalence rates in Spanish-speaking countries and Latin America [19,20].

Although burnout among health sciences students has been widely studied internationally, evidence in the Latin American context—and specifically in Ecuador—is scarce. Dental education in the country presents unique challenges, including limited access to institutional psychological support, demanding clinical workloads, and early exposure to patient care. Understanding burnout in this context is essential for informing targeted interventions and public-health strategies that are sensitive to local educational and institutional realities.

In this context, the objective of the present study is to determine the prevalence of burnout syndrome and to analyze the associated factors in dental students during their clinical training phase.

**H1** **(primary).** *A higher weekly study/workload (>*30 *h/week) is associated with an increased likelihood of experiencing burnout.*

**H2.**  *Students in more advanced clinical semesters (*9*th–*10*th) have a higher probability of burnout compared to those in the* 8*th semester.*

**H3.**  *Differences in burnout prevalence exist between universities, potentially attributable to curricular and organizational variations.*

**H4** **(exploratory).** *Sex and age may be associated with burnout, although previous evidence is heterogeneous.*

## 2. Materials and Methods

### 2.1. Study Design and Data Collection

A multicenter cross-sectional study was conducted at the University of Guayaquil (UG), Universidad de las Américas (UDLA), and Bolivarian University of Ecuador (UBE) during the 2025–2026 academic year. Prior to data collection, authorization letters were submitted to the academic authorities of each institution, outlining the study’s objectives, procedures, and ethical considerations. The research was approved by the Ethics Committee of Universidad of the Americas (CBE180547648/02/25), in accordance with the ethical principles set forth in the Declaration of Helsinki.

Participation in the study was voluntary. All students provided written informed consent after receiving a clear explanation of the study’s objectives, methodology, potential risks, and benefits. Confidentiality of responses was guaranteed, and participants were informed of their right to withdraw at any time without negative consequences. Inclusion criteria were: (i) students officially enrolled in the 8th, 9th, or 10th semester of Dentistry, (ii) active participation in clinical training during the study period, and (iii) provision of written informed consent. Exclusion criteria included: students on academic leave, those not currently engaged in patient care activities, and incomplete questionnaires. Data were collected using a structured self-administered questionnaire, applied in a controlled academic environment to ensure standardization across the three universities. Only fully completed questionnaires were included in the final analysis; incomplete or inconsistent responses were excluded to preserve data integrity.

### 2.2. Participants and Sample Calculation

The target population consisted of students officially enrolled in the clinical levels corresponding to the 8th, 9th, and 10th semesters of the Dentistry programs at the three participating institutions. A non-probabilistic convenience sampling technique was used. This approach was selected because the complete sampling frame of all eligible students was not available, participation required voluntary informed consent, and recruitment depended on institutional authorization and access to academic schedules. Convenience sampling allowed efficient inclusion of students actively engaged in clinical training across the three universities, including only those who voluntarily agreed to participate and who adequately completed the administered instruments. The final sample consisted of 312 participants. Sample size was calculated assuming an infinite population due to the unavailability of a complete sampling frame. Maximum variability was assumed in the proportion of individuals affected by burnout syndrome; thus, a conservative estimate of 50% was used, with a maximum admissible margin of error of 5.55%, as detailed below.

(1) $$n=\frac{Z^{2}p(1-p)}{e^{2}}=\frac{{\left(1.96\right)}^{2}(0.5)(0.5)}{{\left(0.0555\right)}^{2}}=311.79\approx 312$$

### 2.3. Research Instruments

Two previously validated and widely used instruments in the literature on mental health and burnout syndrome were employed for data collection:

- Maslach Burnout Inventory (MBI): A 22-item questionnaire that assesses three core dimensions of burnout syndrome: emotional exhaustion, depersonalization, and personal accomplishment. Each item is rated on a Likert-type frequency scale. The total score allows classification of participants into three categories: no risk (0–43 points), at risk (44–87 points), and presence of burnout syndrome (>88 points) [21,22]. This questionnaire was validated by Carlotto in Brazil [23].
- Burnout-Associated Symptom Questionnaire: A complementary instrument consisting of 14 items designed to assess the frequency of physical and emotional symptoms such as chronic fatigue, insomnia, headaches, neck pain, digestive discomfort, and temporomandibular joint (TMJ) pain. A 7-point Likert scale (ranging from 0 to 6), where 0 indicates “never” and 6 indicates “always,” was used to estimate the students’ symptom burden [21,24,25].

Before the start of the study, a pilot test was conducted with 30 students randomly selected from the three participating universities to assess item comprehension and estimate the average response time (approximately 10 min). This procedure also served as a feasibility test of the instruments and data collection process. Feedback received during this phase led to minor wording adjustments to improve clarity, without altering the original structure of the items. In this sample, the Maslach Burnout Inventory (MBI) demonstrated high internal consistency (*α* = 0.89 overall), with *α* = 0.86 for emotional exhaustion, α = 0.81 for depersonalization, and α = 0.83 for personal accomplishment. The Physical and Emotional Symptoms Questionnaire also showed strong reliability (*α* = 0.87).

### 2.4. Statistical Analysis

The analysis of the results was conducted through a comprehensive approach encompassing descriptive, associative, inferential, and predictive dimensions. First, the descriptive analysis characterized the sample in terms of sociodemographic variables and assessed burnout prevalence, as well as the preliminary exploration of relationships between key variables. From an inferential perspective, Chi-square tests were applied to identify significant associations between qualitative variables, such as academic semester and university of origin. Given that the outcome was dichotomous (presence/absence of burnout), a binary logistic regression model was implemented with two primary objectives: (i) to contrast the predefined hypotheses (H1–H3) and explore H4, evaluating the influence of factors such as age, sex, university, semester, and weekly study/workload on the presence of burnout, and (ii) to estimate adjusted odds ratios (OR) with 95% confidence intervals and p-values, thereby determining whether these factors act as potential risk or protective elements while controlling for confounding in a cross-sectional design with multiple covariates. Additionally, the discriminative capacity of the logistic model was assessed through the Receiver Operating Characteristic (ROC) curve and the calculation of the Area Under the Curve (AUC), allowing for the evaluation of the model’s effectiveness in classifying cases with and without burnout. A significance level of *α* = 0.05 was applied. All analyses were performed using R software version 4.2.1 through the RStudio IDE (Posit, PBC, Boston, MA, USA).

## 3. Results

The sample consisted of 312 dental students enrolled in comprehensive clinical training programs across three Ecuadorian universities. The majority of participants were between 20 and 27 years old (89.74%), followed by 9.29% aged 28 to 35, 0.32% aged 36 to 42, and 0.64% over 42 years of age, indicating a predominantly young population. Regarding sex distribution, 67.63% of the participants were female and 32.37% were male. In terms of institutional affiliation, 73.40% of students were from the University of Guayaquil, 20.19% from the University of the Americas, and 6.41% from the Bolivarian University of Ecuador. As for academic level, participants were enrolled in the eighth (20.19%), ninth (50.32%), and tenth (29.49%) semesters. No students from the seventh semester were included, indicating that the sample was focused on advanced stages of clinical training (Table 1).

In the emotional exhaustion dimension, 79.49% of participants exhibited high levels, 17.63% moderate levels, and 2.88% low levels, indicating a predominant emotional overload, albeit with some variability (SD = 7.21). In the depersonalization dimension, 54.49% reported high levels, 40.06% moderate levels, and 5.45% low levels, suggesting that more than half of the students experience some degree of emotional detachment (SD = 3.50). Regarding personal accomplishment, 70.19% showed high levels, 18.27% moderate levels, and 11.54% low levels. Although the majority maintain a positive perception of their performance, a subgroup at potential risk of decline in this area was identified (SD = 4.60) (Table 2).

By applying the established diagnostic criteria, high levels of emotional exhaustion and depersonalization combined with low personal accomplishment, it was found that 8.01% (*n* = 25) of the students presented clinically evident burnout syndrome. The remaining 91.99% did not meet the criteria for this condition (Table 3).

**Figure 1:**
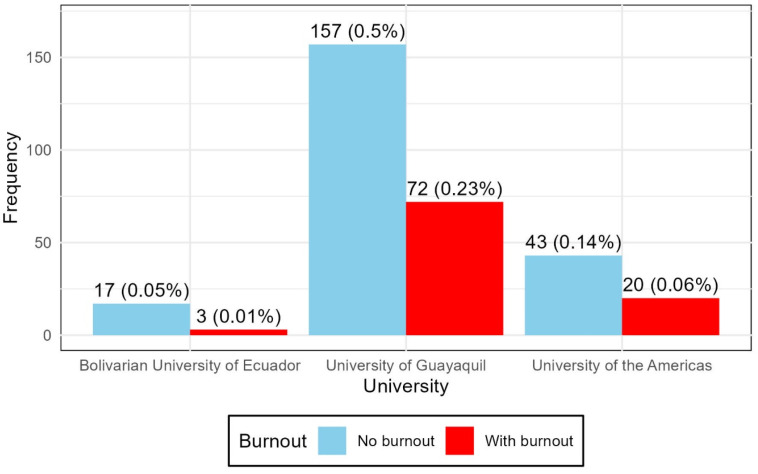
Presence of Burnout syndrome at the university.

The distribution of burnout by university is shown in Figure 1. No statistically significant association was observed between university and burnout (*χ*^2^ = 2.4105; *df* = 2; *p* = 0.2996). (Figure 1).

Despite these percentage differences, the Chi-square test did not reveal a statistically significant association between university and the presence of burnout syndrome (*χ*^2^ = 2.4105; *df* = 2; *p* = 0.2996). Similarly, no significant differences were observed when comparing the presence of the syndrome with the academic semester (*χ^2^* = 0.072; *df* = 2; *p* = 0.9646), nor between men and women (*χ*^2^ = 1.151; *df* = 1; *p* = 0.2834) (Table 4).

To explore factors associated with burnout, a logistic regression model was estimated. The model showed a significant intercept (*β* = −2.747; *p* = 0.003), but none of the independent variables reached statistical significance. Regarding study and work hours, students reporting more than 30 h per week exhibited a higher odds ratio for burnout (OR = 3.39; 95% CI: 0.41–21.88; *p* = 0.208), although this was not statistically significant. The group reporting 21 to 30 h per week also showed a non-significant trend (OR = 1.29; 95% CI: 0.28–6.02; *p* = 0.742). Concerning academic level, ninth-semester students had an OR of 1.99 (95% CI: 0.66–7.37; *p* = 0.252), and tenth-semester students had an OR of 1.31 (95% CI: 0.37–5.30; *p* = 0.683), both non-significant. In the comparison by university, students from the University of Guayaquil showed an OR of 0.81 (95% CI: 0.19–5.55; *p* = 0.796), and those from the University of the Americas showed an OR of 0.09 (95% CI: 0.00–1.10; *p* = 0.067), both compared to Bolivarian University of Ecuador (Table 5).

**Figure 2:**
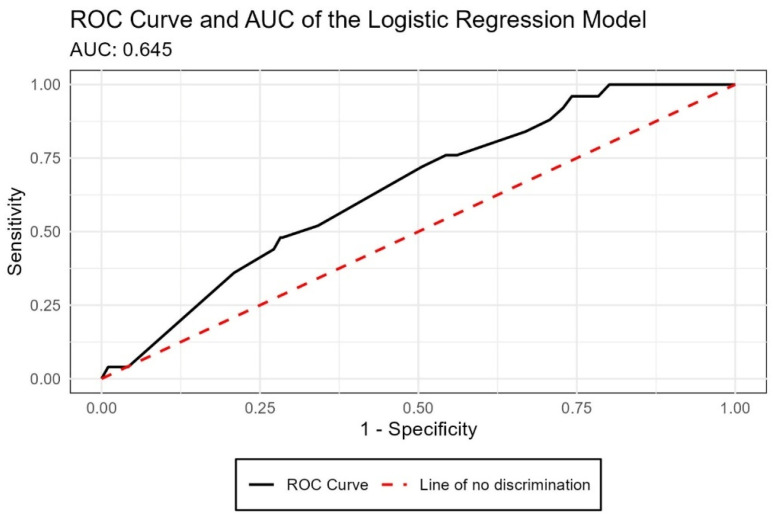
ROC curve and AUC of the logistic regression model for predicting burnout syndrome.

The predictive capacity of the logistic regression model was evaluated using the ROC curve. The area under the curve (AUC) was 0.645, indicating a moderately low discriminative ability. Practically speaking, the model has a 64.5% predictive capacity to correctly identify the presence of burnout syndrome, which limits its clinical or institutional utility (Figure 2).

Regarding the symptom burden reported by dental students, a high prevalence of physical discomfort was observed, particularly in the musculoskeletal domain (Table 6). Frequent or constant lower back and waist pain was reported by 41.35% of participants, followed by neck pain at 35.9% and headaches at 30.45%. These findings reflect a notable presence of musculoskeletal symptoms possibly related to sustained postures, prolonged clinical demands, and accumulated tension during practical activities. On the emotional level, 42.31% of students reported a decreased ability to enjoy daily activities, while 39.42% reported constant fatigue, suggesting compromised psychological well-being likely associated with emotional exhaustion. Additionally, a significant prevalence of somatic symptoms such as digestive problems (28.85%) and limb pain (23.72%) was observed, which may represent physical manifestations of psychological distress. Conversely, certain symptoms were less prevalent. For example, 34.94% of participants reported never experiencing nausea or vomiting, and 37.5% did not report respiratory problems, indicating that these symptoms are not part of the predominant symptomatic profile in this population (Table 6).

Table 7 shows the associations between physical and psychological symptoms and the three dimensions of burnout: emotional fatigue, depersonalization, and personal fulfillment. Emotional fatigue was significantly associated with most symptoms, including somatic and behavioral complaints, while depersonalization was primarily linked to physical symptoms. Personal fulfillment showed fewer significant associations, mainly with enjoyment of activities, temporomandibular joint pain, fatigue, and breathing problems. Overall, emotional fatigue was the most affected dimension, followed by depersonalization, whereas personal fulfillment was less influenced.

## 4. Discussion

The results of this study show a prevalence of 8.01% of burnout syndrome (BS) among dental students in clinical training in Ecuador. However, particularly high levels of emotional exhaustion (79.5%) and depersonalization (54.5%) were observed, while low personal accomplishment affected 11.5% of students. These findings are consistent with international research reporting elevated levels of stress and burnout among health sciences students, especially in dentistry, due to high academic demands, clinical pressure, and early responsibilities during training [26,27]. A systematic review conducted in 2018 by Alzahem et al. [28] indicates that although the prevalence of burnout varies across countries, it generally presents with high levels of emotional exhaustion and depersonalization, closely linked to academic workload. Similarly, Dyrbye et al. (2020) report that students in health-related fields, including dentistry, exhibit high rates of stress and burnout, attributable to intense academic demands, clinical pressure, and early professional responsibilities [29].

When contextualizing our findings, the prevalence of clinically defined burnout in our sample (8.0%) is notably lower than that reported in other student groups. A meta-analysis estimated a pooled prevalence of 37.2% (95% CI 32.7–42.1) in medical students [30], and another review found rates ranging from 7.0% to 75.2% depending on country, instrument, and cutoff criteria [31]. These variations include reports as high as 88% in some contexts [32]. In general non-student working populations, a systematic review of Swiss workers estimated clinical or severe burnout at 4%, with overall burnout and emotional exhaustion both at 18% [33].

The high incidence of burnout syndrome (BS) may be attributed to multiple factors: constant interaction with patients, the need to make critical clinical decisions, and institutional conditions such as lack of psychological support, limited resources, and the perception of inadequate preparation. In this context, studies such as that of Bhattacharyya et al. [34] have identified that participation in extracurricular activities can act as a protective factor against burnout by reducing emotional exhaustion and depersonalization. This evidence is consistent with research highlighting the positive role of such activities in supporting students’ psychological well-being [35,36,37,38].

The high prevalence of emotional exhaustion has also been reported in other similar studies [12,39,40], although broader systematic reviews have shown variability in burnout levels attributable to differences in educational contexts, the use of technology, and pedagogical resources [41,42]. These data suggest that academic training in dentistry is closely related to the development of burnout, highlighting the need to revisit the structural and pedagogical approaches used in the country. Despite the high prevalence observed, modifiable factors were also identified that could help reduce the impact of burnout syndrome. The implementation of institutional programs focused on psychological well-being, adjustment of academic workload, and promotion of extracurricular activities emerges as a key strategy to mitigate emotional exhaustion and enhance students’ educational experience. Another important finding was the association between the number of hours dedicated to study/work per week and levels of exhaustion. Students who reported spending more than 30 h per week had a higher odds ratio for burnout (OR = 3.39; 95% CI: 0.41–21.88; *p* = 0.208). In the university comparison, students from the University of Guayaquil showed an OR of 0.81 (95% CI: 0.19–5.55; *p* = 0.796), and those from the University of the Americas had an OR of 0.09 (95% CI: 0.00–1.10; *p* = 0.067), both compared to the Bolivarian University of Ecuador. It was also observed that students in their ninth or tenth semester reported higher levels of exhaustion, possibly due to the added responsibilities, both professional and personal, that typically emerge during the final stage of the program [43]. These findings underscore the need to design targeted interventions that take into account both workload and the stage of training. A comprehensive approach that addresses academic, institutional, and personal factors will be essential to improving the mental health and academic performance of the country’s future dental professionals. On the other hand, the findings of this study reveal a high prevalence of musculoskeletal complaints among dental students, with lower back pain (41.35%) and neck pain (35.9%) being the most frequently reported symptoms—findings consistent with those reported in several studies, such as Srivastava et al. (2021) [44]. It emphasizes that psychological and genetic factors, along with inadequate physical postures, are key contributors to the development of musculoskeletal disorders (MSDs), which tend to worsen with increased duration of professional dental practice factors that likely contribute to the symptomatology observed in our study population. Moreover, regarding emotional aspects, 42.31% of students reported a reduced ability to enjoy everyday activities, and 39.42% reported experiencing constant fatigue—symptoms that are also linked to psychological burnout and chronic stress, as noted by Sezer et al. (2022) [45]. The coexistence of somatic symptoms, such as digestive problems (28.85%) and limb pain (23.72%), reinforces the evidence that psychological distress can manifest as physical symptoms—a phenomenon widely documented in the literature (Hashim et al., 2021; Rocha CO et al., 2017) [46,47].

Another relevant aspect is that the logistic regression model showed moderate-to-low predictive capacity (AUC = 0.645). This indicates that sociodemographic and academic variables such as sex, age, semester, university, and workload are insufficient to reliably predict burnout. These results highlight the multifactorial nature of the syndrome and suggest that future studies should incorporate personal factors (e.g., coping strategies, resilience, mental health history) and institutional aspects (e.g., curriculum design, academic environment, access to psychological support). While the model may not function as a screening tool, it underscores the limitations of conventional predictors and the need for more holistic prevention and intervention strategies. Importantly, the absence of statistically significant predictors should not be seen as a weakness but rather as a meaningful finding: it shows that traditional variables alone cannot explain the occurrence of burnout. Reporting these null results helps avoid publication bias and directs future research toward underexplored psychological and institutional factors. In this way, the study contributes to advancing knowledge by clarifying what does not predict burnout and by pointing to the need for broader and more comprehensive explanatory models.

From a public-health perspective, these findings emphasize the importance of implementing institutional measures such as routine mental health screening, accessible psychological support services, adjustment of academic workloads, and integration of ergonomic and occupational health training into clinical practice. At a broader level, coordinated actions between universities and health authorities are recommended to establish surveillance systems, allocate resources for preventive programs, and design curricular reforms that prioritize student well-being. Such strategies would not only help reduce burnout and related musculoskeletal complaints but also contribute to the long-term health and professional performance of future dentists.

Finally, despite the limitations inherent to its cross-sectional design, this study provides a comprehensive overview of burnout and its associations with physical and emotional symptoms. The use of validated questionnaires ensured reliable data collection, and the inclusion of students from three representative universities supports generalizability to Ecuador’s dental student population. Overall, the careful selection of participants and robust analysis strengthen the conclusions and provide valuable evidence to guide institutional policies and support programs aimed at improving student well-being.

### 4.1. Contributions

#### 4.1.1. Theoretical Contributions

This study provides new evidence about burnout among dental students in Ecuador, a context with scarce prior data. Psychometric testing confirmed the high internal consistency of the Maslach Burnout Inventory (*α* = 0.89), supporting its use in this population. Although 79.5% of students reported high emotional exhaustion and 54.5% reported high depersonalization, only 11.5% had low personal accomplishment, resulting in a moderate clinical prevalence of burnout (8.01%). This dissociation highlights that burnout dimensions do not necessarily co-occur at the same intensity. Moreover, classical predictors such as semester, sex, or university showed no significant associations, and the logistic regression model demonstrated limited predictive capacity (AUC = 0.645). These findings suggest that future research should incorporate personal traits (e.g., resilience, coping styles) and institutional factors (e.g., workload policies, faculty support) to build more comprehensive explanatory models.

#### 4.1.2. Practical Contributions

On a practical level, our results emphasize the need for universities to implement targeted interventions. First, the weak predictive capacity of traditional variables indicates that prevention should be universal rather than selective. Specific strategies include: (i) adjusting clinical and academic workloads to avoid sustained exposure beyond 30 h/week, since this group showed a higher odds ratio for burnout (OR = 3.39, *p* = 0.208); (ii) integrating ergonomics and posture training into dental curricula, given the high prevalence of musculoskeletal pain (41.3% low back, 35.9% neck, 30.5% headaches); and (iii) offering accessible psychological support services and routine mental health screening with validated tools such as the MBI. At a national level, these findings can inform policies to incorporate systematic monitoring of student well-being into dental education, ensuring both academic performance and long-term professional health.

## 5. Conclusions

This study demonstrates that burnout syndrome affects a significant proportion of dental students in their clinical stage, highlighting elevated levels of emotional exhaustion and related physical symptoms. Although no statistically significant associations were identified with academic or institutional variables, the perceived emotional burden reflects a demanding educational environment that may compromise student well-being. These results provide a better understanding of the scope of burnout in this specific context and pave the way for future research incorporating personal, emotional, and contextual variables to address this issue more comprehensively.

## 6. Limitations and Areas for Future Research

This study has some limitations that should be acknowledged. First, the use of a cross-sectional design prevents establishing causal relationships between academic or personal factors and burnout. Second, the convenience sampling strategy, although pragmatic and common in educational research, limits the generalizability of results to all dental students in Ecuador. Third, data collection relied on self-administered questionnaires in academic settings, which may have been influenced by recall bias or social desirability bias (e.g., students providing responses they perceived as favorable). Although confidentiality was assured and validated instruments were employed to minimize these risks, such potential sources of bias should be considered when interpreting the findings. Finally, the analysis included only academic and sociodemographic predictors; psychological traits (e.g., resilience, coping strategies) and institutional factors (e.g., faculty support, curricular design) were not assessed. Future research should address these limitations by using longitudinal designs to explore the progression of burnout over time, probability-based sampling to improve representativeness, and mixed-method approaches that combine quantitative measures with qualitative insights from students. Expanding the analysis to include personal and institutional variables, as well as evaluating the effectiveness of interventions such as workload adjustments, mental health programs, and ergonomic training, will provide a more comprehensive understanding of burnout and inform strategies to better support dental students in Ecuador and similar contexts.

## Figures and Tables

**Table 1:** Sociodemographic Analysis of the Sample of Dental Students.

Variable	*n* (%)
Age	
20 to 27	280 (89.74)
28 to 35	29 (9.29)
36 to 42	1 (0.32)
Over 42	2 (0.64)
Sex	
Male	101 (32.37)
Female	211 (67.63)
University	
Bolivarian University of Ecuador	20 (6.41)
University of Guayaquil	229 (73.40)
University of the Americas	63 (20.19)
Semester	
Eighth	63 (20.19)
Ninth	157 (50.32)
Tenth	92 (29.49)

**Table 2:** Distribution of burnout levels according to dimension evaluated.

Level	Emotional Exhaustion *n* (%)	Depersonalization *n* (%)	Personal Accomplishment *n* (%)
High	248 (79.49)	170 (54.49)	219 (70.19)
Medium	55 (17.63)	125 (40.06)	57 (18.27)
Low	9 (2.88)	17 (5.45)	36 (11.54)
Estimated Mean (SD)	28.64 (7.21)	10 (3.50)	30.10 (4.60)

**Table 3:** General classification of students according to the presence of burnout syndrome.

Presence of Burnout	*n* (%)
No Burnout	287 (91.99)
Burnout Present	25 (8.01)
Total	312

**Table 4:** Association test between university and semester and the presence of burnout syndrome.

Relation	Statistical	Gl	*p*-Value
Burnout vs. University	2.4105	2	0.2996
Burnout vs. Semester	0.072	2	0.9646
Burnout vs. sex	1.151	1	0.2834

**Table 5:** Logistic model for predicting the presence of Burnout syndrome.

Variable	*β*	SE	*Z*	*p*-Value	OR	95% CI (Lower–Upper)
Intercept	−2.747	0.908	−3.015	0.003	0.06	0.01–0.31
Hours of study/work						
11–20 h (vs. ≤10 h)	0.296	0.598	0.495	0.620	1.34	0.45–4.99
21–30 h	0.251	0.761	0.330	0.742	1.29	0.28–6.02
More than 30 h	1.222	0.970	1.260	0.208	3.39	0.41–21.88
Semester						
Ninth (vs. Eighth)	0.687	0.600	1.144	0.252	1.99	0.66–7.37
Tenth (vs. Eighth)	0.270	0.661	0.409	0.683	1.31	0.37–5.30
University						
University of Guayaquil (vs. Bolivarian University of Ecuador)	−0.210	0.814	−0.258	0.796	0.81	0.19–5.55
University of the Americas (vs. Bolivarian University of Ecuador)	−2.374	1.30	−1.832	0.067	0.09	0.00–1.10

**Table 6:** Prevalence of physical symptoms among dental students.

Symptoms	Never *n* (%)	Few Times *n* (%)	Sometimes *n* (%)	Frequently *n* (%)	Always *n* (%)
**Enjoy activities**	20 (6.41)	67 (21.47)	132 (42.31)	68 (21.79)	25 (8.01)
**Headaches**	7 (2.24)	34 (10.9)	93 (29.81)	83 (26.6)	95 (30.45)
**Neck pain**	15 (4.81)	22 (7.05)	87 (27.88)	76 (24.36)	112 (35.9)
**Temporomandibular joint (TMJ) pain**	86 (27.56)	70 (22.44)	74 (23.72)	39 (12.5)	43 (13.78)
**Back and waist pain**	7 (2.24)	17 (5.45)	86 (27.56)	73 (23.4)	129 (41.35)
**Pain in the extremities of the body**	37 (11.86)	52 (16.67)	88 (28.21)	61 (19.55)	74 (23.72)
**Fatigue with ease**	17 (5.45)	74 (23.72)	123 (39.42)	53 (16.99)	45 (14.42)
**Digestive problems**	41 (13.14)	74 (23.72)	90 (28.85)	46 (14.74)	61 (19.55)
**Loss of appetite**	47 (15.06)	63 (20.19)	109 (34.94)	47 (15.06)	46 (14.74)
**Increased appetite**	36 (11.54)	88 (28.21)	91 (29.17)	49 (15.71)	48 (15.38)
**Nausea and vomiting**	109 (34.94)	79 (25.32)	74 (23.72)	28 (8.97)	22 (7.05)
**Trembling hands**	54 (17.31)	63 (20.19)	101 (32.37)	38 (12.18)	56 (17.95)
**Breathing problems**	117 (37.5)	80 (25.64)	64 (20.51)	21 (6.73)	30 (9.62)

**Table 7:** Association of physical symptoms among dimensions of Burnout syndrome.

Symptoms	Emotional Fatigue			Depersonalization			Personal Fulfillment		
	*χ^2^*	*df*	*p*	*X^2^*	*df*	*p*	*χ^2^*	*df*	*p*
**Enjoy activities**	25.277	8	0.001	12.974	8	0.113	22.433	8	0.004
**Headaches**	31.587	8	0.000	21.294	8	0.006	4.145	8	0.844
**Neck pain**	35.226	8	0.000	23.613	8	0.003	4.494	8	0.810
**Temporomandibular joint (TMJ) pain**	20.857	8	0.008	13.936	8	0.083	15.757	8	0.046
**Back and waist pain**	74.629	8	0.000	29.255	8	0.000	11.169	8	0.192
**Pain in the extremities of the body**	40.941	8	0.000	21.182	8	0.007	10.288	8	0.245
**Fatigue with ease**	54.120	8	0.000	24.039	8	0.002	17.061	8	0.029
**Digestive problems**	31.986	8	0.000	40.360	8	0.000	9.262	8	0.321
**Loss of appetite**	32.255	8	0.000	34.590	8	0.000	11.712	8	0.165
**Increased appetite**	15.266	8	0.054	3.788	8	0.876	8.008	8	0.433
**Nausea and vomiting**	26.935	8	0.001	18.818	8	0.016	7.647	8	0.469
**Trembling hands**	38.998	8	0.000	37.680	8	0.000	4.034	8	0.854
**Breathing problems**	29.224	8	0.000	34.646	8	0.000	21.056	8	0.007

## Data Availability

Data is contained within the article.
